# Racial Differences in Post-Acute Utilization After Hip Fracture in Medicare Beneficiaries With ADRD

**DOI:** 10.1093/geroni/igaa057.192

**Published:** 2020-12-16

**Authors:** Indrakshi Roy, Amol Karmarkar, Amit Kumar, Meghan Warren, Patricia Pohl, Maricruz Rivera-Hernandez

**Affiliations:** 1 Northern Arizona University, flagstaff, Arizona, United States; 2 Virginia Commonwealth University Health, Richmond, Virginia, United States; 3 Northern Arizona University, Flagstaff, Arizona, United States; 4 Northern Arizona University, Phoenix, Arizona, United States; 5 Brown University School of Public Health, Providence, Rhode Island, United States

## Abstract

BACKGROUND: The incidence of hip fracture in patients with Alzheimer’s disease and related dementias (ADRD) is 2.7 times higher than it is in those without ADRD. Care complexity, including extensive post-acute rehabilitation, increases substantially in patients with ADRD after hip fracture. However, there are no standardized post-acute care utilization models for patients with ADRD after hip fracture. Additionally, there is a lack of knowledge on how post-acute utilization varies by race/ethnicity, in this population. OBJECTIVES: To investigate racial differences in post-acute care utilization following hip fracture related hospitalization in patients with ADRD. METHODS: A secondary analysis was conducted on 120,179 older adults with ADRD with incident hip fracture, using 100% Medicare data (2016-2017). The primary outcome was post-acute discharge dispositions (skilled nursing facility [SNF], inpatient rehabilitation facility [IRF], and Home Health Care [HHC]) across various racial groups. Multinomial logistic regression examined the association between race and post-acute discharge dispositions after accounting for patient-level covariates. RESULTS: Compared to non-Hispanic Whites, minority racial groups have significantly lower odds of being discharged to SNF, IRF, or HHC, as compared to home. Adjusted odds ratio for Hispanics discharged to SNF was 0.28 (CI=0.24-0.31), to IRF was 0.46 (CI=0.39-0.52) and HHC was 0.64 (95% CI =0.54-0.75), as compared to home. CONCLUSION: ADRD patients have higher risk of hip fracture. Findings from this study will provide insight on how to reduce racial and ethnic disparities in post-acute care utilization in vulnerable populations and improve quality of care and health outcomes.

